# The Effect of In Vitro Gastrointestinal Digestion on the Antioxidants, Antioxidant Activity, and Hypolipidemic Activity of Green Jujube Vinegar

**DOI:** 10.3390/foods11111647

**Published:** 2022-06-02

**Authors:** Guifeng Li, Ni Yan, Guoqin Li

**Affiliations:** College of Food Science, Shanxi Normal University, Taiyuan 030031, China; 18334810506@163.com (N.Y.); guoqin.li1@uq.net.au (G.L.)

**Keywords:** green jujube vinegar, antioxidants, antioxidant activity, hypolipidemic activity, in vitro gastrointestinal digestion

## Abstract

Healthy fruit vinegar has been extensively favored in China in recent years. As a new type of fruit vinegar developed by our laboratory, green jujube vinegar has the characteristics of good taste and rich nutrition. To study the effect of in vitro gastrointestinal digestion on the antioxidant and hypolipidemic activity of green jujube vinegar, so as to provide basic data for research and the development of healthy food antioxidants, including the total phenolic content (TPC), total flavonoid content (TFC), total acid content, and volatile acid content, were measured. The antioxidant activity was measured by using 2,2-Diphenyl-1-picrylhydrazyl (DPPH) and 2,2′-Azino-bis(3-ethylbenzthiazoline-6-sulfonic acid) (ABTS) free radical scavenging methods and the ferric reducing antioxidant power assay (FRAP), and the hypolipidemic activity was measured by cholesterol adsorption and the sodium cholate adsorption capacities. The results show that gastric digestion significantly (*p* < 0.05) decreased the TPC, TFC, total acid content, and volatile acid content, for which the highest reductions were up to 54.17%, 72%, 88.83% and 82.35%, respectively. During intestinal digestion, the TFC remained at a high level and unchanged, and the TFC and volatile acid content significantly (*p* < 0.05) decreased by 72.66% and 89.05%, respectively. The volatile acid content did not significantly (*p* > 0.05) change within 2 h. The ABTS free radical scavenging ability and the reducing power free radical scavenging rate were correlated with the TPC, TFC, and total acid contents, and the DPPH free radical scavenging ability and cholesterol adsorption capacity were not. These findings suggest that green jujube vinegar can be a potential functional food for people’s use.

## 1. Introduction

Jujube (*Ziziphus jujuba Mill*), a fruit of the buckthorn family (*Rhamnaceae*), is extensively cultivated in the subtropical hilly regions of the world, especially in China, India, and North Africa [1]. More than 400 cultivars in China have been grown for more than 4000 years [2]. It plays a crucial part in human health, due to its abundant bioactive compounds, including vitamin C, phenolic acids, flavonoids, organic acids, ascorbic acid, and mineral constituents. The bioactive compounds can protect against different diseases through their bacteriostasis, and antioxidative, hypolipidemic, antihyperglycemic, and anti-obesity pathways [3,4,5].

The maturity stages of jujube can be divided into three stages—white maturities, half-red maturity, and red maturity. Fruit at the white maturity stage is green jujube. Green jujube has a high level of phenolic content and free radical scavenging (DPPH) and ferric reducing abilities (FRAP) [6,7]. Therefore, choosing green jujube as a raw material to develop new products can solve jujube’s stability issues, enrich the deep processing products of jujube, and bring economic benefits to local enterprises.

Vinegar is known as the “fourth-generation beverage”. It is produced from fruits or fruit-processing wastes and is mainly brewed by alcoholic and acidic fermentation [8]. Vinegar is also rich in bioactive compounds, which play antioxidant, antitumor, anticarcinogenic, and antibacterial roles in maintaining human health [9,10]. Bioactive compounds mainly include phenolics, flavonoids, Vitamin C, and volatile and non-volatile acids. However, gastrointestinal digestion can influence antioxidants’ functional properties. Therefore, the stability of antioxidants in in vitro gastrointestinal digestion is an important indicator that can reflect their possible beneficial influences on human health [11]. The physicochemical factors, such as pH, enzymes and temperature, can affect the bioavailability of substances under gastrointestinal conditions. In vitro gastrointestinal digestion methods have been commonly used to estimate the stability and absorbability of antioxidants in recent years [12,13].

It is generally known that the antioxidant activity of fruit vinegar is related to its contents of bioactive compounds, such as phenolic acids and flavonoids. However, these compounds’ chemical properties and functions might be altered by the chemical reactions during gastrointestinal digestion, which can cause different antioxidant results. In addition, it is not comprehensive to use only one method to evaluate the antioxidant capacity. Since antioxidant activity is determined by the interaction of different mechanisms, it is important to combine multiple methods to measure the antioxidant activity of foods in vitro [14]. Therefore, the antioxidant activity was determined by three different methods in this study to estimate the changes caused by in vitro digestion.

In recent years, hyperlipidemia has become a common public disease. It can induce coronary atherosclerotic heart disease, fatty liver, etc., which is extremely harmful to human health. Thus, the prevention and treatment of hyperlipidemia are extremely important. Natural hypolipidemic drugs have drawn public attention due to their low costs and fewer side effects. An increase in human cholesterol content is an important factor leading to hyperlipidemia. Cholesterol is decomposed in the human body to produce cholate. The adsorption of cholate can promote the catabolism of sodium cholate, thereby reducing the cholesterol content and achieving the goal of the hypolipidemic activity. Therefore, the adsorption of cholesterol and sodium cholate content can be used as an important indicator to measure hypolipidemic activity [15].

To the best of our knowledge, no studies have been published on the changes to the antioxidants and hypolipidemic activities in green jujube vinegar during in vitro gastrointestinal digestion. In this study, the antioxidants, including total phenolic (TP), total flavonoid (TF), total acid, and volatile acid content, were determined. The antioxidant activity was obtained by different methods (FRAP, DPPH, and ABTS), and the hypolipidemic activity was evaluated by cholesterol adsorption and sodium cholate adsorption capacities.

## 2. Materials and Methods

### 2.1. Materials

#### 2.1.1. Chemicals

Sodium chloride (NaCl, 99%), potassium chloride (KCl, >99%), sodium hydrogen carbonate (NaHCO_3_, >99%), calcium chloride (CaCl_2_, >98%), hydrochloric acid (HCl, 37%), ammonium chloride (NH_4_Cl), sodium dihydrogen phosphate monohydrate (NaH_2_PO_4_·H_2_O, >99%), potassium dihydrogen phosphate (KH_2_PO_4_), magnesium chloride (MgCl_2_, >99%), urea (≥99%), potassium persulfate (K_2_S_2_O_8_, >99%), potassium ferricyanide, and trichloroacetic acid (TCA) were obtained from Shanghai Macklin Biochemical Co. Ltd. (Shanghai, China). Folin–Ciocalteu reagent, pepsin (from porcine gastric mucosa), mucin (from the porcine stomach), pancreatin (from the porcine pancreas), lipase (from the porcine pancreas), bile extract porcine, 2-diphenyl-1-picrylhydrazyl (DPPH, 95%), and 2,2′-azinobis (3-ethylbenzthiazoline-6-sulfonic acid) (ABTS, >98%) were obtained from Sigma-Aldrich.

#### 2.1.2. Food Samples

Green jujubes were collected from a local orchard in Linfen, China.

### 2.2. Green Jujube Vinegar Preparation

After removing the jujube pits, the green jujubes were incubated in a water bath at 90 °C for 10–15 min at a fruit-to-water ratio of 1:2. Then, 0.3% pectinase was added and hydrolyzed at 40 °C for 3 h. Green jujube juice was obtained by juicing the fruits with the laboratory juicer (Kaijie Instrument Manufacturing Co., Ltd., Shanghai, China) and then straining them through gauze.

Alcoholic fermentation: The dry yeast was activated with 2% sugar water at 33~38 ℃ for 15~20 min. After adjusting the initial sugar content to 24%, adding 0.3% yeast and 80 mg/L SO_2_, the green jujube wine was fermented at 23 ℃ for 8 days.

Acidic fermentation: 200 mL of green jujube wine was accurately absorbed, and then 10% acetic acid bacteria were added and the wine was sealed with gauze. The acetic acid bacteria solution was obtained by shaking the wine at 32 ℃ and 160 r/min for 24 h. The alcohol content (15%) of the green jujube wine was adjusted to 6% with distilled water, and then 8% activated acetic acid bacteria were added. Finally, the wine was fermented at 32 ℃ for 7 days to get green jujube vinegar.

### 2.3. Simulated Gastric and Intestinal Digestion In Vitro

In vitro gastrointestinal digestion of the samples was performed according to the assay described by Flores et al., with some modifications [16]. The compositions of the simulated gastrointestinal digestive juices are shown in Table 1. Green jujube vinegar (4 mL) was taken without any treatment and was used as a sample for gastric digestion for 0 h. Gastric juice (10 mL) was added to the gastric digestion group, while 1 mol/L HCl solution was added to the gastric acid control group to adjust the pH to 1.30 ± 0.02. No treatment was conducted on the control group. After 2 h of gastric digestion, 10 mL of duodenal and 4 mL of bile juices were added to the intestinal digestion group, while 1 mol/L NaHCO_3_ solution was added to adjust the pH to 8.0 ± 0.2 as the intestinal control group. The sample was incubated in a water bath at 37 °C and was shaken during all the processes. The samples were taken at 0, 0.5, 1, 1.5, and 2 h from both stages and then centrifuged (15 min, 1800× *g*). After the supernatants were collected, the infusions were all frozen at −20 °C for further analysis.

### 2.4. Determination of Antioxidants Content

The total phenolic content (TPC) was measured by the Folin–Ciocalteu assay [1]. Samples of H_2_O (0.2 mL and 2.4 mL) were transferred to test tubes, to which 0.5 mL of undiluted Folin–Ciocalteu reagent was added subsequently. After 2 min, 1.0 mL of 7.5% (*w*/*v*) Na_2_CO_3_ and 0.9 mL of H_2_O were added. After 1 h incubation at room temperature and in the dark, the absorbance was measured at 760 nm.

The total flavonoid content (TFC) was determined according to the methods of Lima et al. [17]. Briefly, 2 mL of the samples were mixed with 1 mL of NaNO_2_ solution (50 g/L). After 6 min, 1.5 mL of AlCl_3_ solution (100 g/L) was added. The mixture was allowed to react for a further 6 min. Subsequently, 4 mL of NaOH solution (200 g/L) and anhydrous ethanol (60%, *w*/*v*) were added to the mixture until a final volume of 25 mL was reached. After standing for 15 min, the absorbance was measured at 510 nm.

The total acid content was measured according to the method described by the national standards of China, GB/T 12456-2008 (Determination of total acid in foods, in Chinese) (PRC, 2008) [18]. Sample pre-treatment: a minimum sample of 200 mL was placed in a 500 mL beaker. It was placed on an electric furnace, stirred while heating to slightly boiling, boiled for 2 min, and weighed. It was then replenished with boiled water to boil quality and placed in an airtight glass container.

Samples of 10~50 mL were placed in 100 mL beakers. The contents of the beakers were transferred to 250 mL volumetric flasks (for a total volume of about 150 mL) with boiled water at about 80 ℃ and then placed in a boiling water bath to boil for 30 min. The samples were shaken 2~3 times so that all the organic acids were dissolved in the solution. They were then left to cool down to 20 °C, replenished with boiled water to 250 mL, and strained through a filter paper. Then, 25 mL of the above samples were put into 250 mL triangle flasks, and 40 mL of water and 0.2 mL of 1% phenolphthalein indicator were added. The samples were titrated with 0.1 mol/L of NaOH standard solution until the solution was reddish and did not fade for 30 s. At the same time, a blank test was performed. The total acid content was determined as follows:

(1) $$\mathrm{Total}\;\mathrm{acid}\;\mathrm{content}\;(g/100\;\mathrm{mL})\;=\frac{c\times \left(V1-V2\right)\times K\times F}{m}\times 100,$$

where c is the concentration of the standard NaOH solution; V1 is the volume of NaOH solution that the sample titration consumes; V2 is the volume of the NaOH solution that the blank titration consumes; K is the conversion coefficient of acetic acid, 0.06; F is the dilution ratio of the sample solution; m is the sample volume.

The volatile acid content was measured with the following method. Samples of 80~100 mL were placed in triangular flasks and continuously stirred with an electromagnetic stirrer. At the same time, the samples were pumped under a low vacuum for 2~4 min to remove the CO_2_. The above-treated samples (25 mL) were transferred to distillation flasks and added with 25 mL of CO_2_-free distilled water and 1 mL of H_3_PO_4_ solution (10%, *w*/*v*). The steam distillation unit was connected and heated until the distillate was about 300 mL. A blank test was conducted under the same conditions. The distillate was heated to 60~65 ℃, and 3~4 drops of phenolphthalein indicator were added. The samples were titrated with 0.1 mol/L of NaOH standard solution until the solution was reddish and did not fade for 30 s. The volatile acid content was determined as follows:

(2) $$\mathrm{Volatile}\;\mathrm{acid}\;\mathrm{content}\;(g/100\;\mathrm{mL})=\;\frac{V1-V2\times c}{m}\times 0.067\times 100,$$

where m is the sample volume; V1 is the volume of the NaOH solution that the sample titration consumes; V2 is the volume of the NaOH solution that the blank titration consumes; c is the concentration of the standard NaOH solution.

### 2.5. Determination of Antioxidant Activity

A DPPH radical scavenging activity assay was conducted [19]. Briefly, 2 mL of the samples were mixed with 2 mL of DPPH solution (0.1 mmol/L in ethanol), and the absorbance was measured at 517 nm after standing for 30 min in the dark. Anhydrous ethanol was used as the reference solution. The radical scavenger activity was determined as follows:

(3) $$\mathrm{DPPH}\;\mathrm{radical}\;\mathrm{scavenging}\;\mathrm{rate}\;(\%)=1-\frac{\mathrm{Ai}-\mathrm{Aj}}{A0}\times 100,$$

where Ai is the absorbance of 2 mL of the sample and 2 mL of DPPH; Aj is the absorbance of 2 mL of the sample and 2 mL of anhydrous ethanol; A0 is the absorbance of 2 mL of DPPH and 2 mL of anhydrous ethanol.

The ABTS radical scavenging activity was determined as described by Giese, et al. [20], with some modifications. The ABTS^+^· solution was produced by reacting 10 mL of aqueous ABTS solution (7 mmol/L) with 10 mL of potassium persulfate (2.45 mmol/L) and keeping it in the dark at 4 °C for 12~16 h before use. Then, 0.1 mol/L of phosphate buffer solution (pH 7.4) was used to adjust the absorbance of the ABTS^+^·at 734 nm to 0.70 ± 0.02. The reactions were performed by adding 3.8 mL of ABTS^+^ solution to 0.2 mL of each sample solution. After 6 min of incubation at room temperature, the absorbance was measured at 734 nm. Anhydrous ethanol was used as the control. The radical scavenger activity was determined as follows:

(4) $$\mathrm{ABTS}\;\mathrm{radical}\;\mathrm{scavenging}\;\mathrm{rate}\;(\%)=1-\frac{A1-A3}{A2}\times 100,$$

where A1 is the absorbance of 0.2 mL of the sample and 3.8 mL of the ABTS working liquid; A2 is the absorbance of 0.2 mL of anhydrous ethanol and 3.8 mL of the ABTS working liquid; A3 is the absorbance of 0.2 mL of the sample and 3.8 mL of anhydrous ethanol.

The reducing power was evaluated as described by [21]. Briefly, 0.5 mL of the sample was added into 1.25 mL of pH 6.6 phosphate buffer solution (0.2 mol/L) and 1.25 mL of potassium ferricyanide (1%, *w*/*v*). The mixture was incubated at 50 ℃ for 20 min, then added to 2.5 mL of trichloroacetic acid solution (1%, *w*/*v*) and centrifuged at 3000 r/min for 10 min. Then, 0.25 mL of ferric chloride (0.1%, *w*/*v*) and 1.25 mL of distilled water were added to the 1.25 mL of supernatant. After the mixture was incubated for 3 min at room temperature, the absorbance was determined at 700 nm. A higher absorbance indicates greater reducing power.

### 2.6. Determination of Hypolipidemic Activity

The cholesterol adsorption capacity was determined according to the method described by Wu et al. [7]. The fresh egg yolk was mixed with distilled water at a ratio of 1:9. The mixture was magnetically stirred for 10 min until emulsified. The egg yolk solution (50 g) was accurately weighed into a conical flask, and 1 mL of the 10-fold diluted sample was added. The mixture was shaken in a water bath at 37 ℃ for 2 h and then centrifuged at 4000 r/min for 20 min. The supernatant (0.4 mL) was added with 0.2 mL of o-phthaladehyde reagent (1 mg/mL) and 4 mL of mixed acid (H_2_SO_4_:acetic acid = 1:1). The absorbance was determined at 550 nm. The cholesterol adsorption activity was determined as follows:

(5) $$\mathrm{Cholesterol}\;\mathrm{adsorption}\;(\mathrm{mg}/\mathrm{mL})=\frac{m1-m2}{v},$$

where m1 is the cholesterol content before adsorption; m2 is the cholesterol content after adsorption; v is the sample volume.

The 10-fold diluted sample (0.5 mL) was added with 100 mL of 0.15 mol/L sodium chloride solution containing 0.2 g of sodium cholate. The solution was shaken in a water bath at 37 ℃ for 2 h, followed by centrifugation at 4000 r/min for 20 min. The supernatant (1 mL) was added with 6 mL of H_2_SO_4_ (45%, *w*/*v*) and 1 mL of furfural (0.3%, *w*/*v*). After the mixture was incubated at 65 °C for 30 min, the absorbance was determined at 620 nm. The sodium cholate adsorption capacity was determined as follows:

(6) $$\mathrm{Sodium}\;\mathrm{cholate}\;\mathrm{adsorption}\;(\mathrm{mg}/\mathrm{mL})=\frac{m3-m4}{v},$$

where m3 is the sodium cholate content before adsorption; m4 is the sodium cholate content after adsorption; v is the sample volume.

### 2.7. Statistical Analysis

A statistical analysis was performed using SPSS Statistics software 22.0. All the experiments were carried out in triplicate, and the data are expressed as the means ± standard deviations (SDs). Significant differences were analyzed by one-way analysis of variance (ANOVA) with Duncan’s test (*p* < 0.05).

## 3. Results and Discussion

### 3.1. Antioxidants

#### 3.1.1. Total Phenolic Content

**Figure 1:**
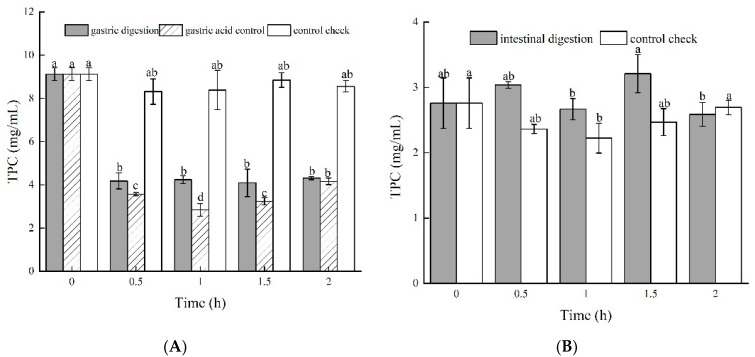

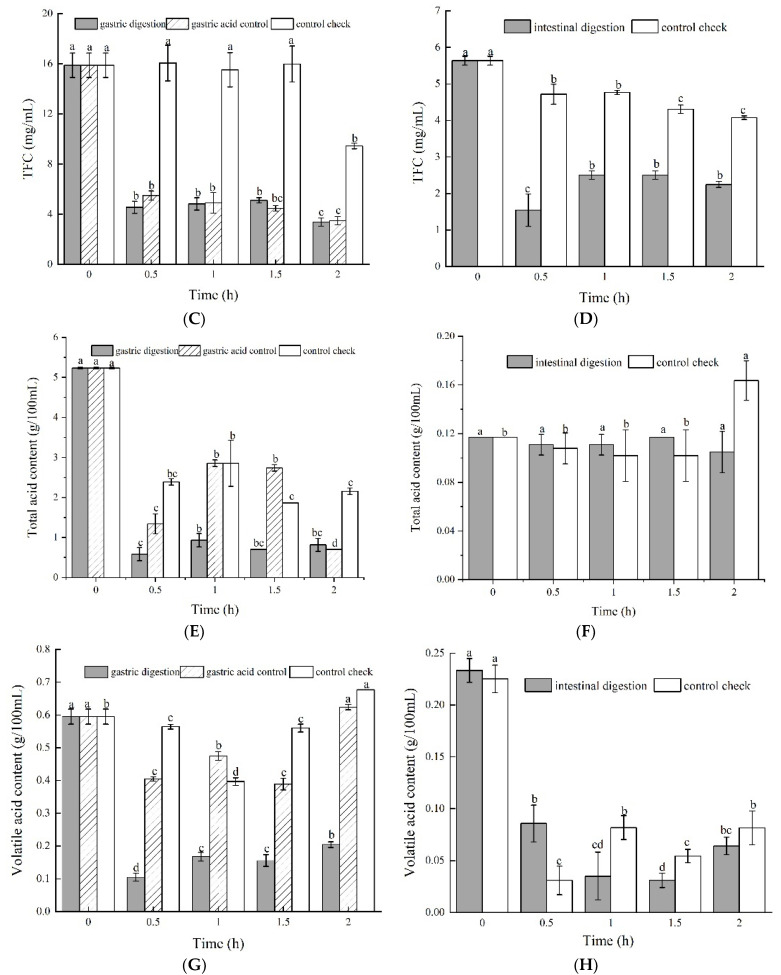
Antioxidants of green jujube vinegar during in vitro gastrointestinal digestion, (**A**,**B**) total phenolic content; (**C**,**D**) total flavonoid content; (**E**,**F**) total acid content; (**G**,**H**) volatile acid content.

The TPC of the control group did not significantly (*p* < 0.05) change within 2 h (Figure 1A). However, the TPC of the gastric acid group decreased by 60.91% within 0.5 h and then increased afterward (Figure 1A). In addition, the TPC of the gastric digestion group decreased by 54.17% within 0.5 h and did not significantly (*p* > 0.05) change afterward (Figure 1A). It is possible that gastric acid degrades phenolic compounds, resulting in a decrease in TPC. However, pepsin can protect phenolic compounds against degradation. These findings are in agreement with the results of Tu et al. [22]. We hypothesize that phenolic compounds might be bound with pepsin, but this needs to be studied further.

The TPC of the intestinal digestion group was significantly (*p* < 0.05) higher than that of the control group at any time before 1 h (Figure 1B). However, the TPC of the intestinal digestion group started to decrease afterward and was significantly (*p* < 0.05) lower than the TPC of the control group. Therefore, the trypsin, lipase, and bile in the intestinal digestion solution might have collaboratively hydrolyzed bound phenolics to be free phenolics and caused the increase in the TPC (Chen et al., 2014) [23]. With increases in the digestion time, the alkaline environment of intestinal digestion may cause the degradation or transformation of phenolic compounds (Carmen et al., 2011; Lima et al., 2019) [17,24].

#### 3.1.2. Total Flavonoid Content

Gastric digestion and gastric acid groups showed similar trends in the total flavonoid content (Figure 1C). The total flavonoid contents of both groups significantly (*p* < 0.05) decreased in the first 0.5 h and did not change with more time. The control group did have not significant changes in the total flavonoid content from 0 to 1.5 h. These findings suggest that gastric acid can decrease the total flavonoid content but pepsin did not have an effect. The intestinal digestion group showed a significantly (*p* < 0.05) lower total flavonoid content than the control group for all durations during the digestion process (Figure 1D). The total flavonoid content decreased by 72.66% during the first 0.5 h, increased slightly at 1 h, and did not change with more time. We can obviously see that intestinal digestion decreased the total flavonoid content mainly at the beginning (0.5 h). The decrease in the total flavonoids in both digestion processes may be a result of flavonoids that are sensitive to pH, which is similar to the findings of Cilla et al. and Zhang et al. [25,26].

#### 3.1.3. Total Acid Content

As shown in Figure 1E, the total acid content of all the groups significantly decreased over time, particularly during the first 0.5 h. The loss in the total acid content is a prominent result of the escape of volatile acid from vinegar samples. The gastric digestion group decreased the total acid content more than the control group. The pH (1.30 ± 0.2) of the gastric digestion solution was close to the pepsin isoelectric point (1–1.25), which led to the decrease in the solubility of the pepsin and the subsequent precipitation. Pepsin, with part of the acid compounds comprising the precipitation, is possibly the main reason for the decrease in the total acid content [27]. Intestinal digestion did not significantly (*p* > 0.05) change the total acid content (Figure 1F).

#### 3.1.4. Volatile Acid Content

As shown in Figure 1G, the volatile acid content of the gastric digestion group decreased more compared to that of the control group, by up to 82.3% in the first 0.5 h. Although the volatile acid content of the gastric digestion group increased gradually after 0.5 h, the change was significantly (*p* < 0.05) lower than that of the control group. We used GC to analyze the composition and content of the volatile acid in the jujube vinegar, and found that acetic acid, butanoic acid, isobutyric acid, and isovaleric acid were mainly included. Pepsin reacting with acetic acid to form precipitation can consume some volatile acid content. Meanwhile, the reaction between salts that occurs in gastric juice and acetic acid also resulted in a decrease in the volatile acid content, which is consistent with the results of Xia et al. [28].

The volatile acid content of the intestinal digestion group decreased by 89.05% from 0.233 g/100 mL to 0.031 g/100 mL in 1.5 h (Figure 1H). The effect of intestinal digestion on decreases in the volatile acid was more obvious than gastric digestion. This implies that enzymes in the intestinal juice can also react with acetic acid and form precipitation, resulting in a decrease in the volatile acid content [29,30]. In addition, the quantity of enzymes in the intestinal juice is much higher than that in the gastric juice, and the decrease in the volatile acid content is larger than that in the gastric juice.

### 3.2. Antioxidant Capacity

#### 3.2.1. DPPH Radical Scavenging Activity

**Figure 2:**
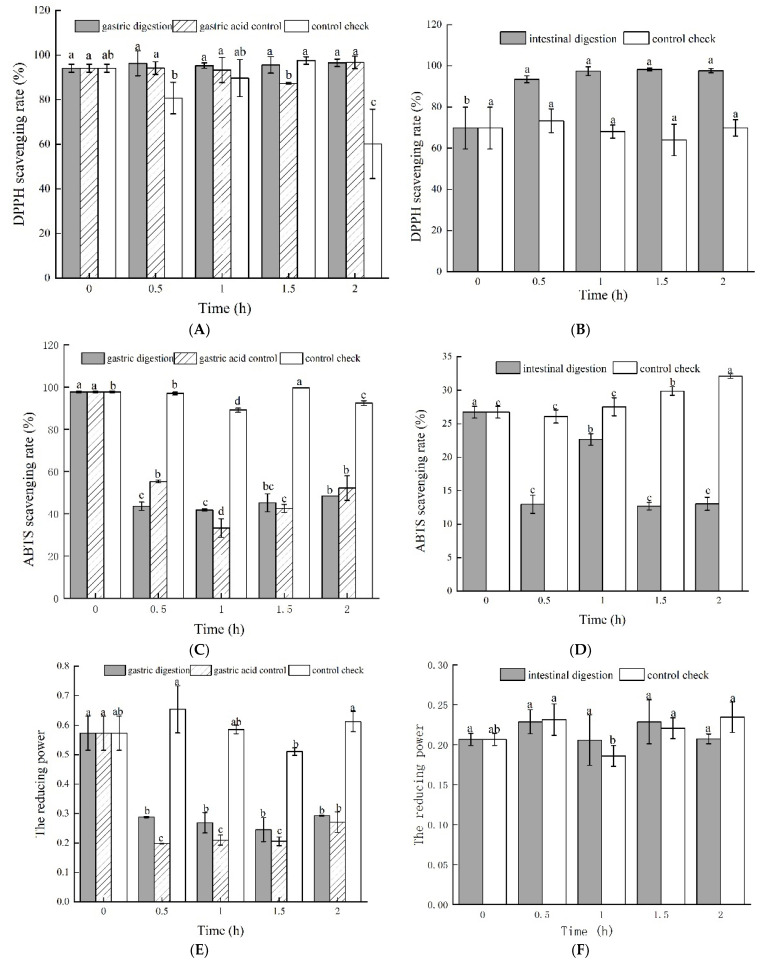
Antioxidant activity of green jujube vinegar during in vitro gastrointestinal digestion, (**A**,**B**) DPPH radical scavenging rate; (**C**,**D**) ABTS radical scavenging rate; (**E**,**F**) reducing power.

The gastric digestion and gastric acid groups exhibited higher DPPH radical scavenging rates than the control group (Figure 2A), although the gastric digestion and gastric acid groups did not significantly (*p* < 0.05) change over time. This implies that pepsin and gastric acid may maintain the DPPH radical scavenging rate.

Intestinal digestion increased DPPH radical scavenging activity by 23.86% in the first 0.5 h, and did not significantly (*p* > 0.05) change after 0.5 h (Figure 2B). However, the control group did not show any significantly different changes over time. This implies that enzymes of the intestinal juice may improve the DPPH radical scavenging rate. It is generally known that the DPPH radical scavenging rate is closely correlated with the content of the total phenolic and flavonoid contents [30,31]. However, the DPPH radical scavenging rate in this study was not significantly correlated with the total phenolic content. The reason is currently not clear and needs to be studied further.

#### 3.2.2. ABTS Radical Scavenging Activity Assay

Although there was no significant (*p* > 0.05) difference between the gastric digestion and gastric acid groups over time, gastric digestion and gastric acid decreased ABTS radical scavenging activity by 50.48% and 46.59%, respectively, during the first 0.5 h, and become relatively stable with more time (Figure 2C). However, the control decreased the ABTS radical scavenging activity less compared to the other groups. We can see that pepsin and gastric acid had an influencing effect on the ABTS radical scavenging activity.

The ABTS radical scavenging of intestinal digestion significantly (*p* < 0.05) decreased within 0.5 h, increased from 0.5 h to 1 h, and remained at a low level after 1 h (Figure 2D). Enzymes and the pH level may be factors that reduce the ABTS radical scavenging activity. However, the control group exhibited a constant increase in the ABTS radical scavenging activity starting at 0.5 h, with a significant difference between 0.5 h and 2 h. A synergistic effect between different active substances is proposed to enhance the ABTS radical scavenging ability, which is consistent with the results of Lu et al. [32] and Masisi et al. [33].

#### 3.2.3. The Reducing Power

The gastric digestion and gastric acid groups showed similar trends in the reducing power (Figure 2E). The reducing power of both groups significantly (*p* < 0.05) decreased in the first 0.5 h and did not change with more time. The reducing power of the gastric digestion group and gastric acid control group decreased by 48.87% and 52.71%, respectively. The reducing power did not significantly change from 0 to 1.5 h in the control group. These findings suggest that gastric acid and pepsin can decrease the reducing power. However, the intestinal digestion and control check groups had no significant difference in the intestinal digestion process (Figure 2F). These results indicate that the reducing power decreased from gastric digestion to intestinal digestion, meaning that the bioactive compound of the green jujube vinegar might have been released due to the pH of the gastric juice containing proteases; the change in the pH value reduced the reducing power. Meanwhile, the hydroxyl groups also affect the reducing power [31].

### 3.3. Hypolipidemic Activity

#### 3.3.1. Cholesterol Adsorption Capacity

**Figure 3:**
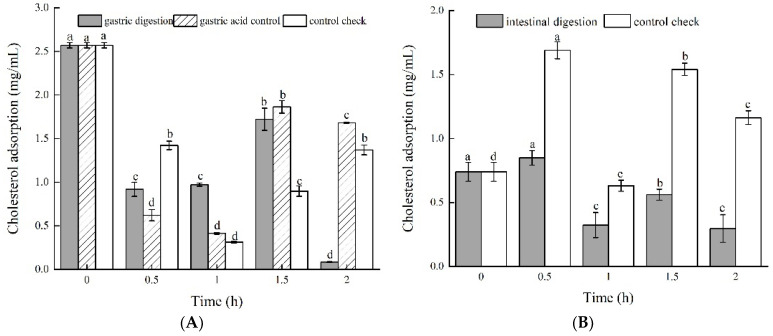

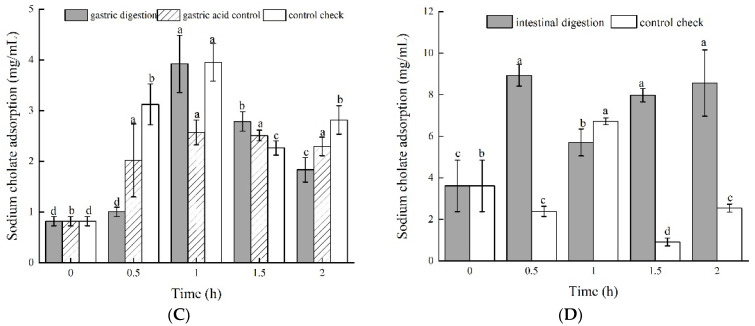
Hypolipidemic activity of green jujube vinegar during in vitro gastrointestinal digestion, (**A**,**B**) cholesterol adsorption; (**C**,**D**) sodium cholate adsorption.

The cholesterol adsorption capacity of green jujube vinegar is shown in Figure 3A,B. In the gastric digestion process, from 0 h to 1 h, the cholesterol adsorption of the three groups decreased gradually, and then subsequently increased up to 0.084 ± 0.006 mg/mL, 1.681 ± 0.006 mg/mL, and 1.370 ± 0.056 mg/mL, respectively (Figure 3A).

Among all the groups, the gastric digestion group was relatively lower than the others. In the intestinal digestion process, the cholesterol adsorption of the intestinal digestion group was significantly lower than that of the control group. At 2 h, the rate of the intestinal digestion group was 0.297 ± 0.108 mg/mL, with a decrease of 59.9%, while the rate of the control group was 1.163 ±0.054 mg/mL, with an increase of 57.2%. Furthermore, inhibitory activity after intestinal digestion was generally higher than that after gastric digestion. These results indicate that the pH and digestive enzymes have greater effects on the cholesterol adsorption capacity of green jujube vinegar [34]. It has been reported that the niacin and other substances in fruit vinegar can promote the excretion of cholesterol in the human body through the intestinal feces and reduce the cholesterol content [35,36]. It may also be caused by various digestive enzymes in the digestive fluid.

#### 3.3.2. Sodium Cholate Adsorption Capacity

The results in Figure 3C,D show the sodium cholate adsorption capacity of the green jujube vinegar during the gastrointestinal simulated digestion process. Compared to the control group, the sodium cholate adsorption capacities of the gastric digestion and gastric acid groups were lower. The gastric digestion group increased rapidly within 0~1 h, and the adsorption capacity reached the highest level at 1 h (3.923 ± 0.563 mg/mL), then gradually decreased, reaching 1.834 ± 0.243 mg/mL at 2 h. The gastric acid group showed the same trend for sodium cholate absorption as the gastric digestion group. In the intestinal digestion process, the sodium cholate adsorption of the intestinal digestion group was much higher than that of the control group, increasing by 1.37-fold from 0 h to 2 h. There was a significant increase from the beginning of the gastric digestion to the ending of the intestinal digestion, which may have been due to the presence of trypsin and lipase in an alkaline intestinal environment [37,38]. However, the specific mechanism needs further study.

### 3.4. Correlation Analysis

Previous studies have reported that there is a high correlation between phenolic compounds and antioxidant activity. Thus, Pearson’s correlation coefficient was used to analyze the correlation between the functional properties of green jujube vinegar after in vitro stimulation (Table 2). ABTS and the reducing power showed extremely significant positive correlations (*p* < 0.01) with the TPC, TFC, total acid, and volatile acid. However, the correlation between the DPPH and the TPC, TFC, total acid, and volatile acid was not significant. These results are different from those of Gullon et al. [31], who reported that DPPH free radical scavenging activity is significantly related to the content of polyphenols and flavonoids, which may be due to the different types of raw materials, maturity, and processing methods.

With regard to the hypolipidemic capacity, cholesterol adsorption presented a positive and very high correlation to TPC (*r* = 0.818), TFC (*r* = 0.854), total acid (*r* = 0.812), volatile acid (*r* = 0.770), ABTS (*r* = 0.773) and the reducing power (*r* = 0.773).

Meanwhile, sodium cholate adsorption showed a significant negative correlation to TFC (*r* = −0.633), ABTS (*r* = −0.819). These results indicate that the antioxidant activity and hypolipidemic activity were strongly influenced by different compounds. However, the specific mechanism needs further study.

## 4. Conclusions

This study presents the effect of in vitro gastrointestinal digestion on the antioxidants, antioxidant activity, and hypolipidemic activity of green jujube vinegar. The TPC, TFC, total acid content, and volatile acid content were all significantly decreased. However, the four parameters showed very different trends during intestinal digestion. The TFC and volatile acid were reduced more than the TPC, but the total acid remained unchanged within 2 h. Gastrointestinal digestion maintained a high DPPH free radical scavenging rate but decreased the ABTS free radical scavenging rate. However, the reducing power in response to gastric and intestinal digestion was varied. The loss of reducing power mostly occurred at the gastric digestion stage. During gastrointestinal digestion, a dramatic increase in the sodium cholate adsorption capacity rather than the cholesterol adsorption capacity can prove that jujube vinegar has strong antioxidant potential and can lower the fat content. Antioxidant activities, including ABTS and reducing power, except DPPH, were found to be significantly (*p* < 0.05) correlated with the TPC, TFC, total acid content, and volatile acid content. The cholesterol adsorption capacity was also found to be significantly (*p* < 0.05) correlated to the TPC, TFC, total acid content, and volatile acid content. These results complete the scale analysis of green jujube vinegar and provide acknowledgment of the stability of functional properties during the digestion process, suggesting that green jujube vinegar is a potentially functional food and could play an important role in hyperlipidemia treatment. Considering the health benefits associated with green jujube vinegar, the application of modern technological processes aims to improve its bioavailability, which is worthy of further research.

## Figures and Tables

**Table 1:** Chemical composition of in vitro digestion juices.

Stock Solutions	Gastric Juice	Duodenal Juice	Bile Juice
Distilled water	500 mL	500 mL	500 mL
NaCl	2.75 g	7.03 g	5.27 g
KCl	0.82 g	0.57 g	0.38 g
NaHCO_3_	-	3.39 g	5.79 g
CaCl_2_·H_2_O	0.40 g	-	-
NaH_2_PO_4_	0.266 g	-	-
KH_2_PO_4_	-	80.30 mg	-
NH_4_Cl	0.306 g	-	-
MgCl_2_	-	50.40 mg	-
Urea	0.09 g	0.10 g	0.26 g
HCl	6.50 mL	0.15 mL	0.15 mL
Adjuncts	2.50 g pepsin, 3.00 g mucin	9.02 g pancreatin, 1.50 g lipase	12.01 g bile salts
pH	1.30 ± 0.02	8.1 ± 0.2	8.2 ± 0.2

**Table 2:** Linear correlation coefficients of functional properties of green jujube vinegar.

	TPC	TFC	Total Acid	Volatile Acid	DPPH	ABTS	Reducing Power	Cholesterol	Sodium Cholate
TPC	1	0.936 **	0.983 **	0.914 **	0.136	0.953 **	0.991 **	0.818 **	−0.631
TFC		1	0.959 **	0.960 **	−0.152	0.914 **	0.930 **	0.854 **	−0.633 *
Total acid			1	0.936 **	0.081	0.921 **	0.987 **	0.812**	−0.550
Volatile acid				1	−0.236	0.907 **	0.914 **	0.770 **	−0.630
DPPH					1	0.046	0.125	−0.037	0.144
ABTS						1	0.927 **	0.773 **	−0.819 **
Reducing power							1	0.773 **	−0.587
Cholesterol								1	−0.477
Sodium cholate									1

** Extremely significant correlation at 0.01 level; * significant correlation at 0.05 level. TPC, total phenolic content; TFC, total flavonoid content.

## Data Availability

Data is contained within the article.
